# Idiopathic intracranial hypertension presenting as iron deficiency anemia: a case report

**DOI:** 10.1186/s13256-020-02631-2

**Published:** 2021-02-02

**Authors:** Peng Yong Sim, Priyal Taribagil, Ione O. C. Woollacott, Safina Rashid, Desmond P. Kidd

**Affiliations:** 1 Department of Ophthalmology, Royal Free Hospital, London, UK; 2 University College London Medical School, London, UK; 3 Department of Neurology, Royal Free Hospital, London, UK

**Keywords:** Iron deficiency anemia, Papilledema, Idiopathic intracranial hypertension, Optic neuropathy, Vision loss, Case report

## Abstract

**Background:**

The presentation of idiopathic intracranial hypertension (IIH) in association with iron deficiency anemia (IDA) is rare.

**Case presentation:**

This case report depicts the unusual case of a 31-year-old woman of mixed Jamaican and English heritage with IIH who presented initially as IDA in the context of menorrhagia. Subsequent ophthalmic review, lumbar puncture, cerebrospinal fluid analysis and neuroimaging studies revealed severe bilateral optic disc swelling and raised intracranial pressure in keeping with IIH. Prompt treatment of IDA with blood transfusion and orally administered iron supplements, in addition to medical treatment for IIH, contributed to significant improvement of symptoms and prevented long-term visual deficits.

**Conclusion:**

The possibility of IDA, albeit rare, should always be considered and investigated appropriately in all patients with IIH, as the treatment of the anemia alone may be sight-saving.

## Background

Idiopathic intracranial hypertension (IIH) is clinically defined as raised intracranial pressure (ICP) without evidence of a detectable cause. Disease prevalence is greatest in obese women of childbearing age, with a global incidence of 12–20 per 100,000 per year in this category. In comparison, the incidence in the general population is only 0.5–2 per 100,000 people per year [1]. IIH was originally referred to as benign intracranial hypertension, but this is now obsolete given its potential to cause severe and permanent blindness [2]. The exact pathogenesis behind IIH remains unknown; however, there is speculation about whether abnormal pressure gradients seen in venous hypertension may contribute to IIH [2].

Iron deficiency anemia (IDA) has been reported as a rare association with IIH. Since its first description in 1840 by Praël [3], fewer than 100 such cases have been reported in the literature [4]. Although cases with isolated IIH and anemia have been described in which full symptomatic resolution was achieved with the treatment of anemia alone [5–10], the causal relationship between these two entities remains questionable. Several mechanisms have been proposed, including a hyperviscous state, which leads to increased venous pressure without true venous sinus thrombosis [4], and a hypoxic state with resultant cerebral edema due to underlying anemia [3].

We present a rare case of IIH presenting initially as IDA in which significant resolution of papilledema was achieved with the prompt and simple correction of anemia, preventing long-term visual deficits. This illustrates the importance of clinician awareness of the rare association of IDA with IIH, especially given that treatment of anemia may be pivotal in optimizing visual outcome.

## Case presentation

**Fig. 1:**
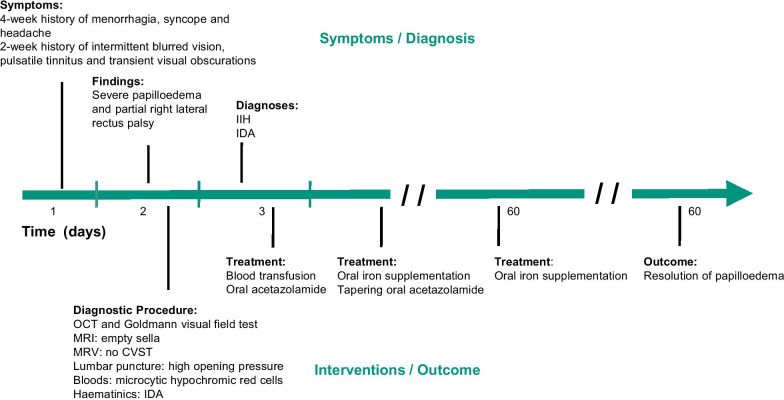
Clinical timeline of a 31-year-old woman of mixed Jamaican and English heritage diagnosed with IIH and IDA. *OCT* optical coherence tomography, *MRI* magnetic resonance imaging, *MRV* magnetic resonance venography, *CVST* cerebral venous sinus thrombosis, *IIH* idiopathic intracranial hypertension, *IDA* iron deficiency anemia

Our patient, a 31-year-old woman of mixed Jamaican and English heritage, first presented to the general accident and emergency department with a 4-week history of menorrhagia and a 3-week history of lethargy, palpitations, exertional dyspnea, postural dizziness, syncopal episodes, headache and vomiting (Fig. 1). She initially denied any visual symptoms and was admitted to the gynecology unit for treatment and monitoring. She had a normal body mass index of 24, and her medical, drug and family history were unremarkable, apart from a congenital left esotropia corrected with bilateral medial rectus recession as a child.

The next day she revealed that over the past 2 weeks she had developed pulsatile tinnitus, intermittent blurred vision and transient visual obscurations. Three days prior to admission she had developed neck pain and photophobia, and 2 days prior to admission mild clumsiness of the right arm and hand and numbness on the right scalp and right fingertips. She underwent an urgent ophthalmic and orthoptic examination, which revealed severe papilledema (grade 5 on Frisén scale) and a partial right lateral rectus palsy. She was afebrile and mildly hypotensive (blood pressure 87/62 mmHg). Best-corrected visual acuity (BCVA) was 6/9 in the right eye and 6/6 in the left eye. Standard color vision test with Ishihara plates was 16/17 in the right eye and 15/17 in the left eye. No relative afferent pupillary defect was demonstrated. Intraocular pressure and anterior segment were unremarkable. The peripheral retina, vessels and other cranial nerves were normal. She also underwent an urgent inpatient neurological review, and peripheral neurological examination was normal.

**Fig. 2:**
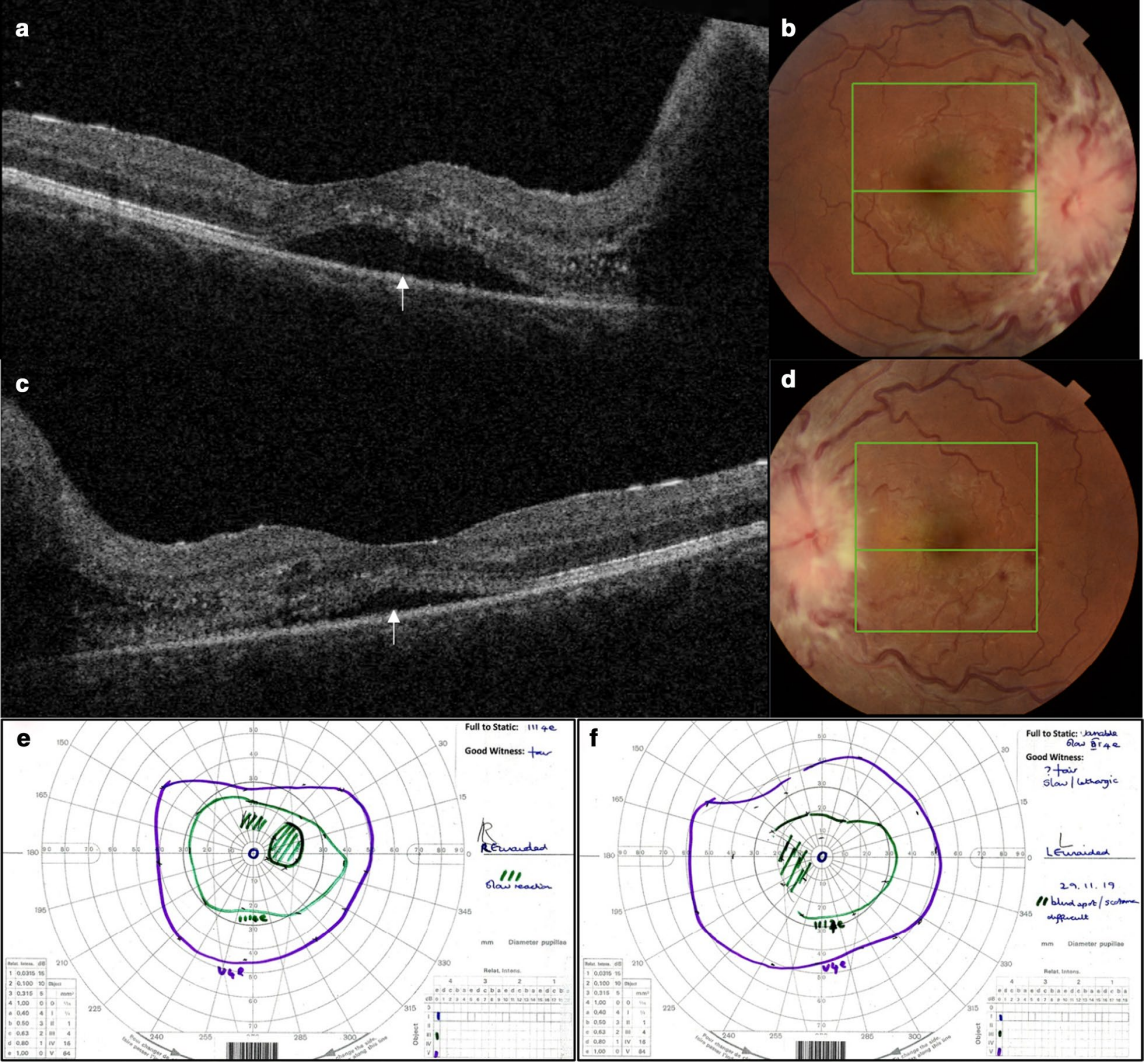
Initial optical coherence tomography (OCT) demonstrating peripapillary and macular subretinal fluid (white arrows). Color fundus photographs (**b** right; **d** left) highlighting the position of the line scan for the OCT images (**a** right; **c** left). Initial Goldmann visual field maps (**e** right; **f** left) showing constricted fields and an enlarged left blind spot

**Fig. 3:**
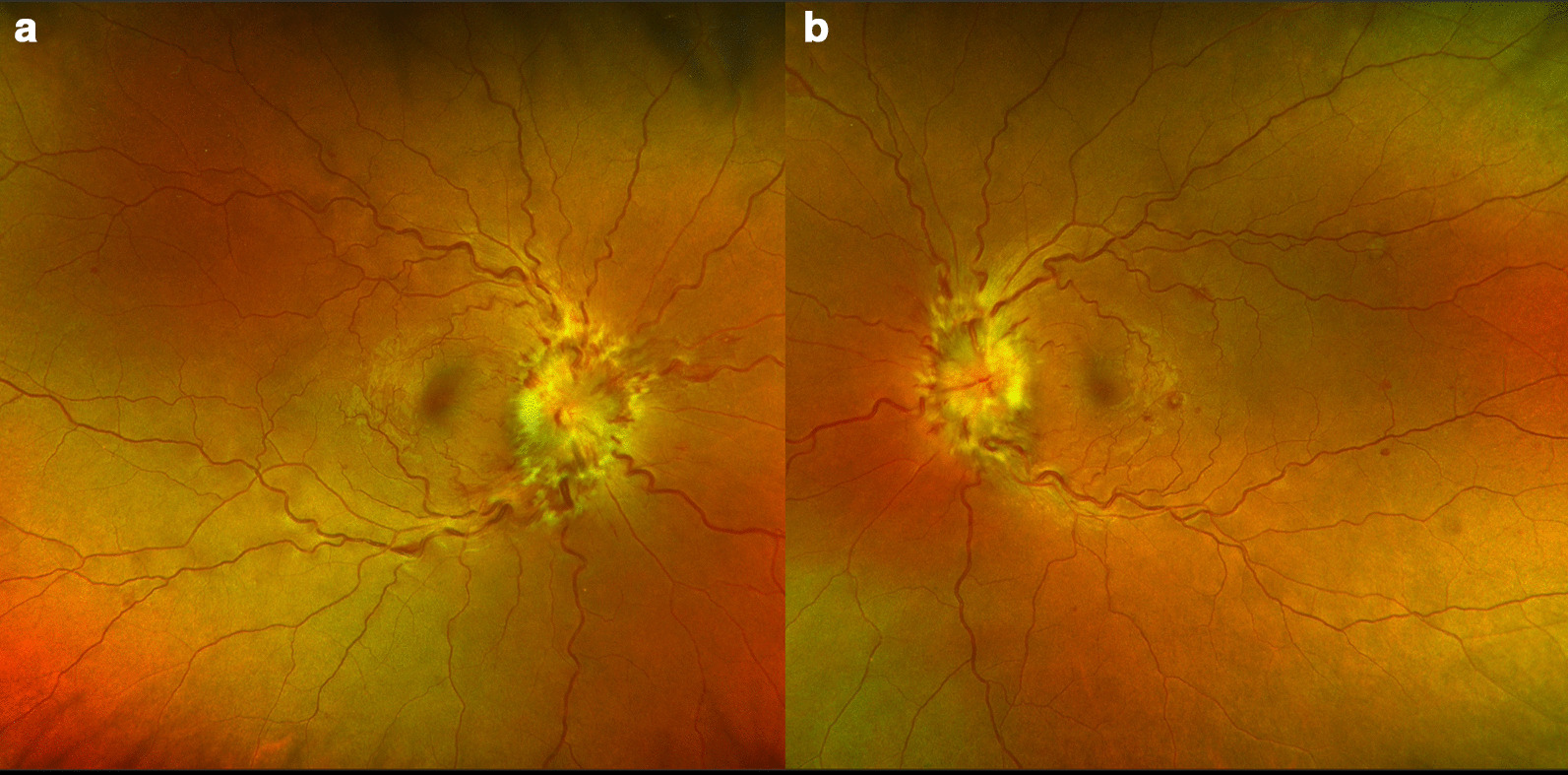
Severe papilledema as demonstrated on Optos ultra-widefield imaging. **a** Right eye. **b** Left eye

Goldmann visual field testing revealed an enlarged left blind spot and constriction of peripheral fields in both eyes (Fig. 2). Ultra-widefield imaging (Optos^®^) confirmed severe papilledema (Fig. 3), and optical coherence tomography (OCT) demonstrated subretinal fluid in the peripapillary and macular area (Fig. 2).

Blood tests revealed a hemoglobin level of 74 gm/L and mean cell volume of 63.0 fL consistent with microcytic anemia, as well as a platelet count of 584 × 10^9^/L consistent with thrombocytosis. Peripheral smear showed microcytic hypochromic red blood cells with anisopoikilocytosis. Iron studies in keeping with iron deficiency were as follows: serum iron 2.2 μmol/L, serum total iron binding capacity 94.7 μmol/L, transferrin saturation 2.3% and ferritin 2 μg/L. The remainder of the results of the full blood count, serum chemistry, liver function, renal function, thyroid function, erythrocyte sedimentation rate, clotting screen, C-reactive protein, vitamin B12 and folate were normal. Transvaginal ultrasound revealed no uterine abnormalities. Comprehensive infection and autoimmune screens were negative. Apart from menorrhagia, no other etiology for IDA was found.

An urgent CT head and CT venogram were performed, which showed no intracerebral lesions but there was bilateral focal narrowing at the transverse and sigmoid junctions, more conspicuous on the left. There were filling defects at the proximal right sigmoid sinus which were felt to be consistent with arachnoid granulations, and there was no evidence of venous sinus thrombosis. There was prominence of the perioptic cerebrospinal fluid (CSF) spaces with minimal flattening of the sclera, especially on the left. CSF opening pressure on lumbar puncture was 63 cm H_2_O and closing pressure 27 cm H_2_O. Cytological and biochemical analyses of CSF were normal. Bacterial culture, viral polymerase chain reaction and malignant cytology were also negative. No other hormonal or metabolic cause of raised ICP was found. Subsequent magnetic resonance imaging (MRI) of the brain with venography revealed findings of bilateral transverse sigmoid sinus narrowing, prominence of the perioptic nerve spaces and an empty sella, consistent with IIH and excluding venous sinus thrombosis.

**Fig. 4:**
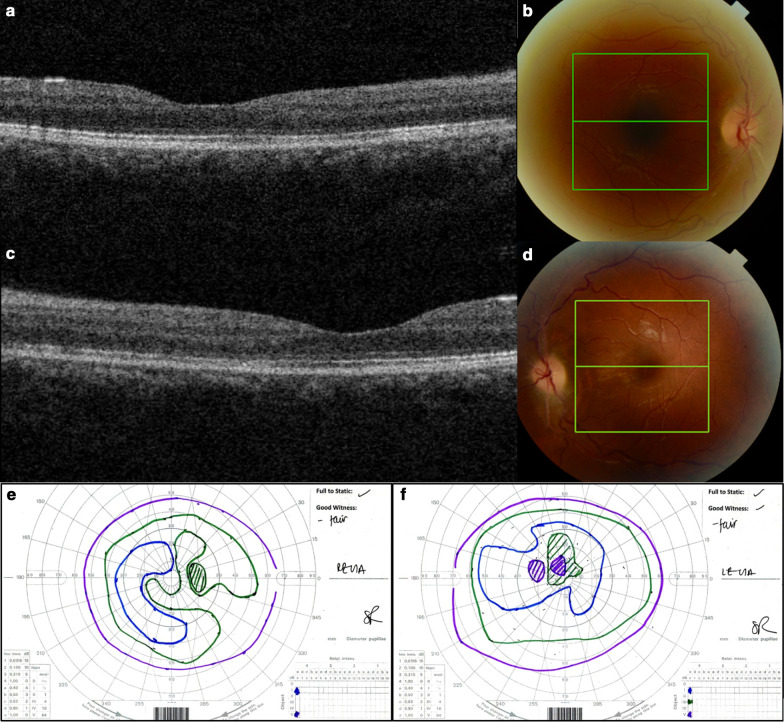
Follow-up optical coherence tomography (OCT) and color fundus photography demonstrating resolution of papilledema. Color fundus photographs (**b** right; **d** left) highlighting the position of the line scan for the OCT images (**a** right; **c** left). Follow-up Goldmann visual field maps (**e** right; **f** left) showing improvement of visual field in both eyes, with a residual central defect in the right eye

The patient was transfused with 2 units of red blood cells and commenced on orally administered iron supplementation and acetazolamide 1 g once a day. All symptoms rapidly improved following this, with return of hemoglobin level to 105 gm/L at day 10. Repeat lumbar puncture at 1-month follow-up revealed a significantly reduced opening pressure of 27 cm H_2_O. There was a gradual resolution of papilledema over the following 2 months (Fig. 4), with preserved BCVA and improved visual field (Fig. 4). Following tapering of acetazolamide treatment, the patient remains asymptomatic whilst solely on iron replacement and is under regular follow-up in our neuro-ophthalmology clinic.

## Discussion and conclusions

Given that IDA is extremely common and is the most prevalent cause of anemia worldwide [11], our case highlights the importance of considering, detecting and treating concomitant IDA in patients with IIH. This is supported by the recent diagnostic criteria for IIH proposed by Friedman and Jacobson that specify severe IDA as a condition that can result in intracranial hypertension and mimic IIH [12]. However, the authors acknowledge that the establishment of a definite association between these two entities is limited by the paucity of reported cases. Our case adds to the growing evidence that IDA is a key etiological factor in a subset of patients with raised ICP, and it may be arguable that such cases should not be termed “true” IIH, but rather secondary intracranial hypertension.

The differential diagnosis of IIH includes masquerading conditions that may produce raised ICP. These include a number of medical disorders (for example, cerebral venous sinus thrombosis, renal failure) and medications (for example, tetracycline, vitamin A and related compounds) [12]. The valid diagnosis of IIH therefore requires the exclusion of secondary causes through a thorough workup, all of which was fulfilled in our case. It remains unclear whether IDA is causative or simply coincidental in the context of our patient with IIH. However, it is likely to be the main etiological factor given the exclusion of all other possible diagnoses with an extensive workup, and significant clinical improvement and maintenance upon normalization of hemoglobin and iron levels. We speculate that the short course of oral acetazolamide acted as a temporizing measure while allowing iron-related homeostatic mechanisms to slowly regain control in normalizing ICP.

Although the causal relationship between IDA and IIH remains speculative [13–15], a recent large retrospective cohort study of 607 patients with IIH found an independent association between IIH and anemia in patients with obesity [16]. This is corroborated by a consecutive case series that found a clinically significant proportion of cases of IDA in patients with IIH [6, 10], as well as observations from isolated case reports where correction of anemia alone corresponded with full resolution of symptoms and signs of IIH [4, 5, 8].

The exact etiological role of anemia in raised ICP remains unclear. Different mechanisms have been posited by different authors, including (1) increased cerebral venous pressure secondary to a relative hyperviscosity state that ultimately leads to raised ICP [4], (2) altered cerebral hemodynamics due to tissue hypoxia which increases brain capillary permeability and therefore ICP [3], and (3) dysfunctional iron hemostasis causing altered CSF dynamics and raised ICP [6].

Of these, the hyperviscosity theory is likely to be the most plausible. IDA is considered a hypercoagulable state and has been associated with venous and arterial cerebral thrombosis [17, 18]. Pathogenic processes in IDA contributing to this may include abnormal blood flow patterns within the vessels as a result of reduced deformability and increased viscosity of microcytic red blood cells [19]. In addition, IDA may result in reactive thrombocytosis due to the lack of inhibition of thrombopoiesis [20, 21]. These processes have been hypothesized to lead to increased venous pressure which decreases the rate of CSF resorption, thereby resulting in raised ICP [4]. Interestingly, cerebral venous sinus thrombosis is often excluded in such cases [4, 6], including our patient, which could suggest IDA-associated hyperviscosity to be a milder variant lying on a similar disease spectrum with true venous sinus thrombosis.

In conclusion, clinicians should be aware of IDA as a rare but important association in patients with IIH. Consequently, the workup of patients presenting with signs of raised ICP should include a full blood count, and if appropriate, hematinics including iron studies, especially in the absence of other known associated factors such as obesity or medications. Prompt evaluation and treatment of IDA should be achieved to reduce, if not prevent, long-term ocular morbidity and mortality.

## Data Availability

Not applicable.

## Abbreviations

CSF: Cerebrospinal fluid
CT: Computed tomography
BCVA: Best corrected visual acuity
ICP: Intracranial pressure
IDA: Iron deficiency anemia
IIH: Idiopathic intracranial hypertension
MRI: Magnetic resonance imaging
OCT: Optical coherence tomography
